# Do stroke services still show sex differences? A multicenter study

**DOI:** 10.1007/s10072-023-07026-x

**Published:** 2023-09-18

**Authors:** Nevine El Nahas, Hossam Shokri, Tamer Roushdy, Noha Dawood, Amr Zaki, Mehdi Farhoudi, Elyar Sadeghi Hokmabadi, Amal Al Hashmi, Waldemar Brola, Krystian Kosno, Cristian Falup-Pecurariu, Bogdan Ciopleias, Joan Montaner, Soledad Pérez-Sánchez, Manoj Mittal, Kandis Dowd, Annie Banke, Nicholas Vigilante, James Siegler, Atilla Ozcan Ozdemir, Ozlem Aykac, Zehra Uysal Kocabas, Donoband Melgarejo, Analia Cardozo, Lorena Peralta, Hany Aref, Valeria Caso

**Affiliations:** 1 Neurology Department, Faculty of Medicine, Ain Shams University, Cairo, Egypt; 2 Neurosciences Research Center, Tabriz University of Medical Sciences, Tabriz, Iran; 3 Central Stroke Unit, Neuroscience Directorate, Khoula Hospital, Muscat, Oman; 4 Department of Neurology, Specialist Hospital Konskie, Collegium Medicum, Jan Kochanowski University, Kielce, Poland; 5 Department of Neurology, Specialist Hospital, Konskie, Poland; 6 Department of Neurology, Faculty of Medicine, University Transilvania, Brasov, Romania; 7 Department of Neurology, County Clinic Hospital, Brasov, Romania; 8 Neurovascular Research Group, Biomedicine Institute of Seville, IBiS/Hospital Virgen del Rocío/CSIC/University of Seville, Seville, Spain; 9 Department of Neurology, Hospital Universitario Virgen Macarena, Seville, Spain; 10 Stroke and Neurocritical Care, Sutter Medical Center, Sacramento, CA USA; 11 Cooper Medical School of Rowan University, Camden, NJ USA; 12 Cooper Neurological Institute, Cooper University Hospital, Camden, NJ USA; 13 Faculty of Medicine, Eskisehir Osmangazi University, Eskisehir, Turkey; 14 Stroke Unit, Instituto de Previsión Social Central Hospital, Asunción, Paraguay; 15 Stroke Unit, Santa Maria Della Misericordia Hospital, University of Perugia, Perugia, Italy

**Keywords:** Stroke services, Stroke, Women, Sex differences, Stroke characteristics, Stroke outcome

## Abstract

**Background:**

The issue of sex differences in stroke has gained concern in the past few years. However, multicenter studies are still required in this field. This study explores sex variation in a large number of patients and compares stroke characteristics among women in different age groups and across different countries.

**Methods:**

This multicenter retrospective cross-sectional study aimed to compare sexes regarding risk factors, stroke severity, quality of services, and stroke outcome. Moreover, conventional risk factors in women according to age groups and among different countries were studied.

**Results:**

Eighteen thousand six hundred fifty-nine patients from 9 countries spanning 4 continents were studied. The number of women was significantly lower than men, with older age, more prevalence of AF, hypertension, and dyslipidemia. Ischemic stroke was more severe in women, with worse outcomes among women (*p*: < 0.0001), although the time to treatment was shorter. Bridging that was more frequent in women (*p*:0.002). Analyzing only women: ischemic stroke was more frequent among the older, while hemorrhage and TIA prevailed in the younger and stroke of undetermined etiology. Comparison between countries showed differences in age, risk factors, type of stroke, and management.

**Conclusion:**

We observed sex differences in risk factors, stroke severity, and outcome in our population. However, access to revascularization was in favor of women.

## Introduction

The incidence of ischemic stroke is increasing with age, especially among women. It ranges from 41.5–70.5/100 thousand in western countries to 131–151.5/100 thousand in eastern countries [1].

Since 2001, the Institute of Medicine has highlighted the importance of studying sex differences in diseases and its impact on outcome [2].

Exploring such differences can promote more precise management protocols in acute and chronic stroke settings. Furthermore, data about sex variability concerning the delay of onset to door and door to treatment times still need unravelling, as this information can impact equity of stroke services and, consequently, stroke outcome [3].

And despite that several articles have demonstrated a minimal sex difference in revascularization therapy yet, this might still negatively influence disease outcomes in women [4, 5].

Moreover, even fewer studies have touched on the issue of stroke in women in the context of an epidemiological study among different geographical regions [6].

Furthermore, the variance in ethnicity, culture, nutrition, and health habits among populations can influence sex-related health services. Thus, stroke risk factors in women need to be investigated in various regions of the world.

Accordingly, the current study was conducted to compare sex differences relevant to stroke risk factors, quality of management, and outcome of acute ischemic stroke (AIS). In addition, we were also interested in exploring these sex differences among various countries.

## Methods

This is a multicentric, observational, cross-sectional, retrospective study done by analyzing stroke units’ databases of participating countries.

Representatives of stroke units/centers who previously participated and co-authored an earlier study [7] were contacted through email and invited to share their stroke units’ data for analysis. Time period was specified in the study protocol sent to all participating centers as “The past 5 years from January 2017 till January 2022.”

A total of 9 centers, representing 9 different countries and 4 continents, participated, including Egypt, Iran, Oman, Poland, Romania, Spain, the USA, Turkey, and Paraguay. All types of stroke were studied (acute ischemic stroke (AIS), hemorrhagic, transient ischemic attack (TIA), and subarachnoid hemorrhage). The study population was evaluated for risk factors, stroke subtypes, stroke severity according to the National Institutes of Health Stroke Scale (NIHSS), and acute stroke management based on current international guidelines: intravenous thrombolysis (IVT), thrombectomy (MT), or bridging between men and women. The outcome was measured by the modified Rankin scale (mRS), defining favorable outcome as mRS < 2.

Women were further divided into the childbearing period (CB) (18–45 years), premenopausal (PM) (46–60 years), and menopausal (M) (> 60 years). A comparison was made between different age categories regarding types of stroke, TOAST classification of ischemic stroke, management, and outcome on discharge and at 3 months follow-up.

Finally, a comparison among countries was made for age, risk factors, type of stroke, and management.

### Statistics

Statistical analysis was done using SPSS version 19^th^ (SPSS Inc., Chicago). To test for normality of continuous data distribution, the Shapiro-Wilks test was used. Mean and standard deviation were used for normally distributed data, while median and interquartile range (IQR) were used for shewed data. Categorical data were presented as frequencies. The Mann–Whitney test or Kruskal–Wallis test was used to compare not normally distributed continuous variables with nominal independent variables. The chi-square test was used for comparison of nominal data.

## Results

### Demographic data and clinical characteristics for both groups: (Table 1)

**Table 1:** Comparison of stroke demographics between both sexes

	Men *n* = 10,437 (55.9%)	Women *n* = 8222 (44.1%)	*p*-value
Age*	65 (57–75)	69 (59–78)	** < 0.0001**
Age category			
(0–45 years) 8.5%	8.6%	8.4%	0.6
(46–60 years) 23.5%	26.6%	19.5%	** < 0.0001**
(> 60 years) 68%	64.7%	72.1%	** < 0.0001**
Atrial fibrillation (admission/past history)	15.6%	20.4%	** < 0.0001**
Hypertension (admission/past history)	68.5%	74.9%	** < 0.0001**
Diabetes (admission/past history)	33.8%	33.7%	0.9
Dyslipidemia (admission/past history)	21.7%	24.3%	** < 0.0001**
Current smoker	22.9%	8.7%	** < 0.0001**
Previous TIA or stroke	16.8%	17.3%	0.33
Congestive heart failure	6.5%	6.9%	0.38
Vascular disease	13.0%	11.8%	**0.02**
NIHSS admission*	8 (3–14)	9 (4–17)	** < 0.0001**
Type of stroke:			
Ischemic stroke	81.1%	79.9%	**0.03**
Hemorrhagic stroke	14.2%	13.3%	0.07
TIA	4%	6%	** < 0.0001**
Subarachnoid hemorrhage	0.7%	0.8%	0.4
Acute intervention/treatment			
Conservative	81.5%	81.3%	0.7
IVT	14.6%	14%	0.2
MT	2.9%	3.3%	0.1
IVT, MT	1%	1.5%	**0.002**
Onset to door (minutes)*	224 (90–720)	180 (83–600)	** < 0.0001**
Door to needle (minutes)*	46 (31–63)	45 (33–62)	0.768
Duration of hospital stay (days) *	6 (3–10)	6 (3–11)	0.185
mRS* discharge (favorable outcome)	29.2%	24.2%	** < 0.0001**
mRS* 3 months (favorable outcome)	39.2%	33.1%	** < 0.0001**

*Median (IQR), *TIA* transient ischemic attack, *NIHSS* National Institutes of Health Stroke Scale, *IVT* intravenous thrombolysis, *MT* mechanical thrombectomy, *mRS* Modified Rankin Scale favorable outcome: (< 2)

Bold values refer to statistically significant variables

#### Risk factors

A total of 18,659 patients were studied most of whom (68%) were in the age group > 60 years. Women constituted 44.1% and had a significantly higher age than men (*p* < 0.0001). The number of women was significantly lower than men in premenopausal age group, and higher in menopausal age group (*p* < 0.0001).

Regarding risk factors, women had more atrial fibrillation (AF), hypertension, and dyslipidemia (*p* < 0.0001). At the same time, they were significantly lower in smoking (*p* < 0.0001) and other vascular diseases (*p* = 0.02).

#### Type of stroke

Ischemic strokes were more frequent in men than in women (*p* = 0.03), while TIA was more in women (*p* < 0.000). There was no difference in hemorrhagic between both sexes.

#### Stroke severity and management

NIHSS was significantly higher in women on admission. Moreover, the outcome, as measured by mRS, was less favorable in women at discharge and after 3 months (*p* < 0.0001). On the other hand, the type of acute management whether conservative or revascularization was similar, except for bridging therapy that was more frequently performed in women (*p* = 0.002).

Onset to door time (OTD) was significantly lower in women (*p* < 0.0001), while door to needle (DTN) and hospital stay showed no difference between the sexes.

### Comparison of stroke characteristics among women: (Table 2)

**Table 2:** Comparison of different age categories among women

	CB	PM	M	*p*-value
Age	18–45 years	46-60 years	> 60 years	CB:PM = < 0.0001
Frequency	*N* = 688	*N* = 1595	*N* = 5891	PM:M = < 0.0001
(%)	(8.4%)	(19.5%)	(72.1%)	CB:M = < 0.0001
*Type of stroke, %*				
Ischemic stroke	67.2%	69.6%	84%	** < 0.0001**
Hemorrhagic stroke	17.3%	16.3%	12.1%	
TIA	12.6%	11.9%	3.75%	
Subarachnoid hemorrhage	2.9%	2.2%	0.2%	
Post hoc	***p*** **-value**			
	CB vs PM	CB vs M	PM vs M	
Ischemic stroke	0.2	** < 0.0001**	** < 0.0001**	
Hemorrhagic stroke	0.5	**0.0001**	** < 0.0001**	
TIA	0.6	** < 0.0001**	** < 0.0001**	
Subarachnoid hemorrhage	0.2	** < 0.0001**	** < 0.0001**	
Type of ischemic stroke				
Small vessel stroke	10.2%	19.9%	14.65	** < 0.0001**
Large vessel disease with stenosis	19.4%	25.5%	23.2%	
Cardio-embolic	20.4%	21%	29.9%	
Undetermined etiology	18.1%	15.1%	12.8%	
Other determined etiology	31.9%	18.4%	19.5%	
Post hoc	***p*** **-value**			
	CB vs M	M vs PM	CB vs PM	
Small vessel/lacunar	**0.001**	** < 0.0001**	** < 0.0001**	
Large vessel disease with stenosis	**0.02**	**0.05**	**0.001**	
Cardio-embolic	** < 0.0001**	** < 0.0001**	0.7	
Undetermined etiology	**0.0001**	**0.01**	0.07	
Other determined etiology	** < 0.0001**	0.3	** < 0.0001**	
Acute intervention treatment				
Conservative	83.8%	82.9%	80.5%	0.065
IVT	12.9%	13.2%	14.3%	
MT	2.1%	2.5%	3.6%	
IVT and MT	1.2%	1.3%	1.6%	
Post hoc	*p*-value			
	CB vs M	M vs PM	CB vs PM	
Conservative	**0.03**	**0.03**	0.6	
IVT	0.3	0.2	0.8	
MT	**0.04**	**0.03**	0.5	
IVT and MT	0.4	0.3	0.8	
Onset to needle (minutes)*	152 (115–190)	150 (109–195)	146 (104–188)	0.533
Door to needle (minutes)*	46 (30.5–85)	46 (35–61)	45 (33–62)	0.776
NIHSS admission	6 (2–13)	7 (3–15)	10 (4–18)	** < 0.001**
mRS discharge (< 2)	43.5%	33.6%	19.9%	** < 0.0001**
Post hoc	CB vs M	M vs PM	CB vs PM	
*p*-value	** < 0.0001**	** < 0.00001**	** < 0.0001**	
mRS 3 months (< 2)	58.5%	46.1%	26.5%	** < 0.0001**
Post hoc	CB vs M	M vs PM	CB vs PM	
*p*-value	** < 0.0001**	** < 0.0001**	** < 0.0001**	

*Median (IQR), *CB* childbearing (18–45 years), *PM* peri-menopause (46–60 years), *M* menopausal (> 60 years), *TIA* transient ischemic attacks, *IVT* intravenous thrombolysis, *MT* mechanical thrombectomy, *mRS* Modified Rankin Scale favorable outcome: (≤ 2)

Bold values refer to statistically significant variables

Women tend to be significantly older (*p* < 0.0001). Ischemic stroke was significantly higher among the older age group (post hoc analysis: menopausal group significantly more than the two younger age groups). On the contrary, hemorrhagic stroke, subarachnoid hemorrhage, and TIA were significantly higher in younger groups (post hoc analysis: the non-significant difference between CB and PM, and significant difference between both groups and M group).

According to TOAST classification, small vessel stroke and large vessel stroke were higher in the PM group (post hoc analysis: significantly higher incidence in PM compared to the other two groups and higher in M compared to CB group). Cardio-embolic stroke was significantly higher in M group as compared to the 2 other younger groups, while there was no difference between CB and PM groups. Stroke of undetermined etiology was higher in younger age (CB) (post hoc analysis: significantly less in M compared to the other two groups). Stroke of other determined etiology was significantly more in CB age group as compared to the other two groups.

Onset to needle and door to needle times showed no disparity among age groups. There was a marginal difference in favor of CB and PM groups compared to M group for conservative management (*p* = 0.03), marginal difference in favor of M group compared with the two younger groups for MT (*p* = 0.04, 0.03), and no difference regarding IVT.

And NIHSS on admission was significantly higher in M group (*p* < 0.001), whereas favorable outcome on discharge and at 3 months was more frequently reported in younger age groups, being more significantly in CB compared to both PM and M and in PM compared to M (*p* < 0.0001).

### Risk Factors according to sex among participating countries: (Table 3)

**Table 3:**
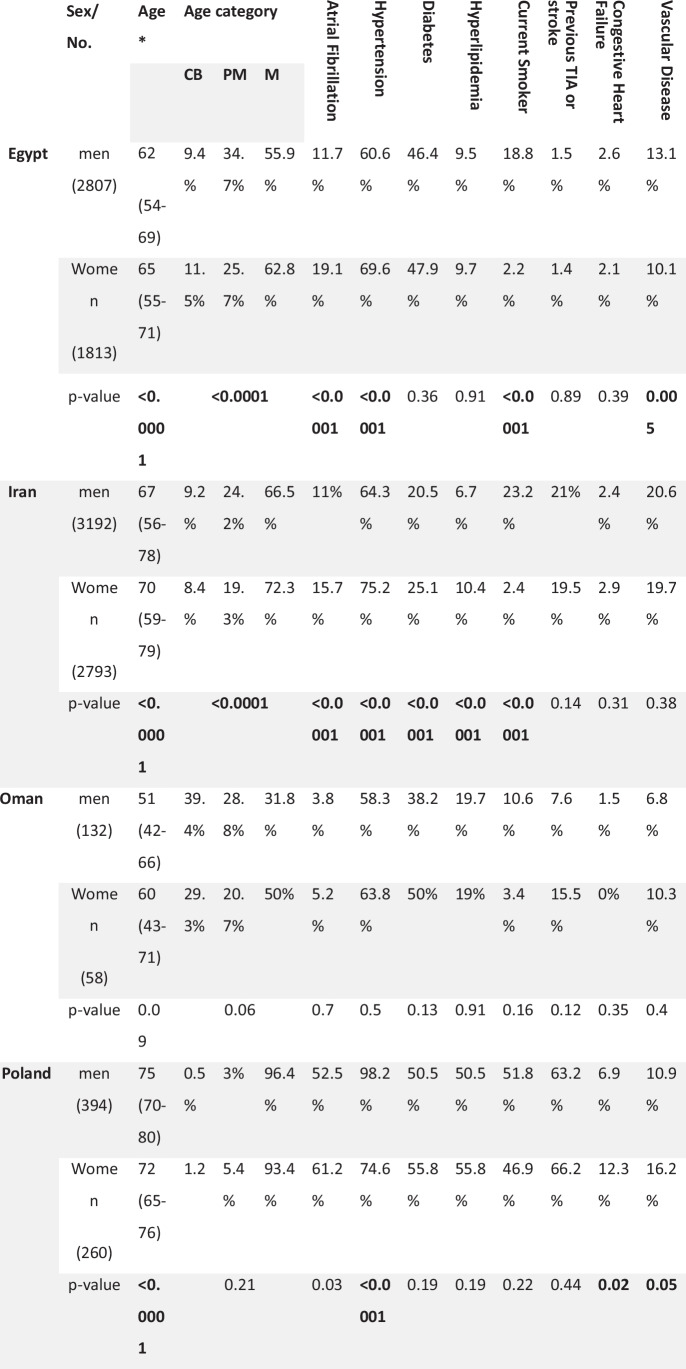

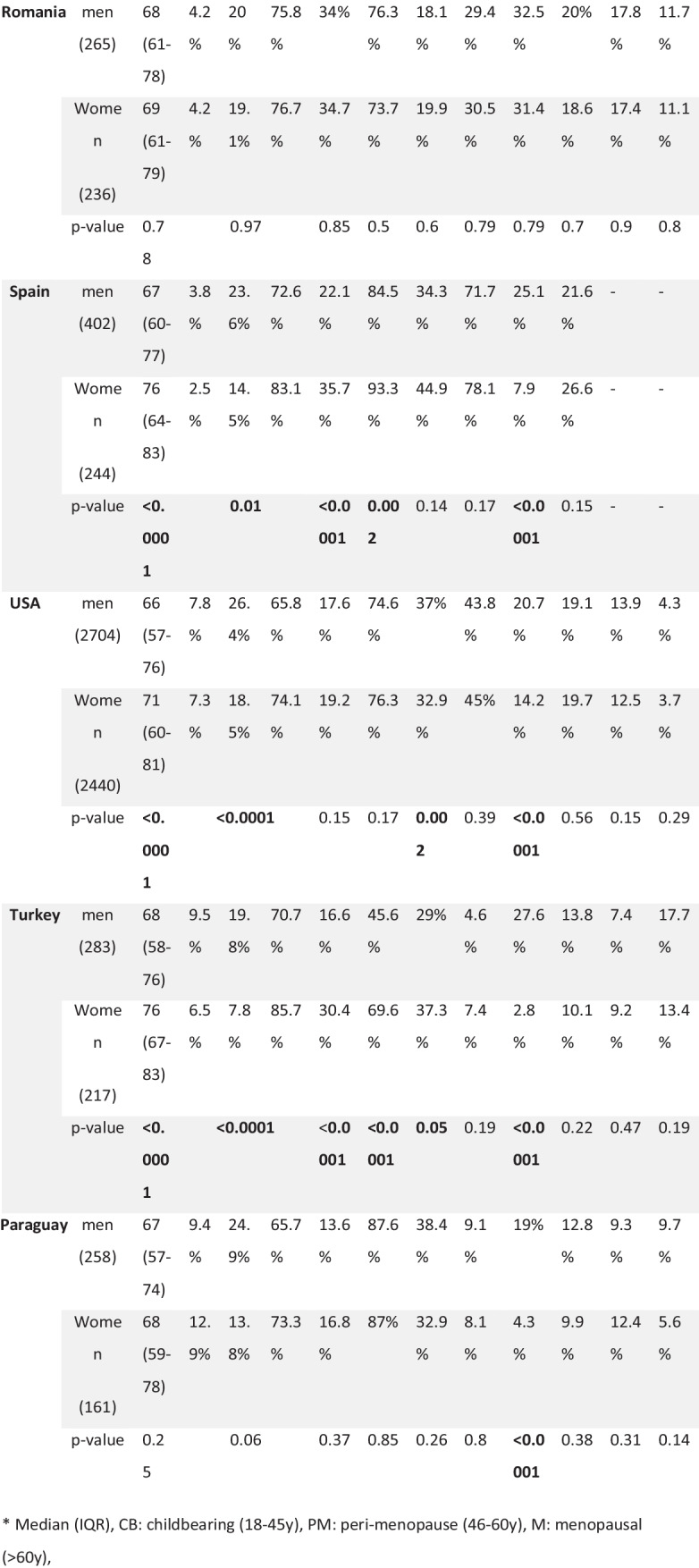
Comparison of risk factors according to sex among the participating countries

* Median (IQR), CB: childbearing (18-45y), PM: peri-menopause (46-60y), M: menopausal (>60y),

The median age in women was lower in both Egypt and Oman as compared to all other countries, and this comes in the context of a younger age of men, as well. It is also notable that the frequency of patients in the 2 younger age groups was higher in these 2 countries (37.2% and 50% respectively) compared to less than 30% in other countries.

AF was significantly more reported among women in Egypt, Iran, Poland, Spain, and Turkey. Hypertension was more reported in women in Egypt, Iran, Spain, and Turkey, and more in men in Poland.

Diabetes was more frequent in women in Iran and Turkey, and more in men in the USA. Dyslipidemia was significantly more frequent in women in Iran only. Smoking was more frequent in men in Egypt, Iran, Spain, USA, Turkey, and Paraguay.

Previous stroke and congestive heart failure showed non-significant difference among both sexes in all countries. And other vascular diseases were more in men only in Egypt.

### Comparison of stroke severity, type, intervention, and outcome among the participating countries: (Tables 4 and 5)

**Table 4:** Comparison of stroke severity, type, and intervention among the participating countries

	Sex/No	NIHSS admission*	*Type of stroke*				*Acute management*				Onset to needle (minutes)*	Door to needle (minutes)*
			Ischemic stroke	Hemorrhagic stroke	TIA	Subarachnoid hemorrhage	Conservative	IVT	MT	IVT, MT		
Egypt	Men (2807)	6 (3–10)	86.6%	7.7%	2.4%	3.3%	82%	16.8%	1%	0.2%	178 (120–210)	45 (30–60)
	Women (1813)	7 (4–12)	85.6%	7.1%	2.4%	5%	82.8%	15.2%	1.2%	0.8%	160 (120–210)	40 (30–60)
							*P:0.4*	*P:0.1*	*P:0.5*	***P:0.002***		
	*p*-value	** < 0.0001**	0.12				**0.008**				0.2	0.2
Iran	Men (3192)	13 (6.3–22)	67.1%	24%	8.9%	-	86.6%	13.3%	-	0.1%	150 (115–195)	45 (33–64)
	Women (2793)	15 (8–25)	65.4%	22.9%	11.7%	-	87.3%	12.6%	-	0.1%	155 (120–198)	48 (37–65)
			*P:0.1*	*P:0.3*	***P:0.004***							
	*p*-value	** < 0.0001**	**0.002**				0.6				0.2	0.07
Oman	Men (132)	6 (3–10)	56.1%	42.4%	0%	1.5%	68.9%	22%	-	9.1%	-	54 (26–66)
	Women (58)	8 (4–12)	73.7%	24.6%	0%	1.8%	49.1%	42.1%	3.5%	5.3%	-	45 (29–64)
			***P:0.02***	***P:0.01***		***P:0.8***	***P:0.009***	***P:0.004***	***P:0.03***	*P:0.3*		
	*p*-value	0.1	**0.05**				**0.003**				NA	0.9
Poland	Men (394)	13 (12–13)	86.3%	13.7%	-	-	85.8%	14%	-	0.3%	150 (135–165)	45 (35–45)
	Women (260)	12 (8–13)	91.9%	7.3%	0.8%	-	62.3%	19.2%	0.4%	18.1%	125 (85–160)	35 (30–45)
			***P:0.02***	***P:0.01***	*P:0.07*		***P:*** ** < ** ***0.0001***	*P:0.07*	*P:0.2*	***P:*** ** < ** ***0.0001***		
	*p*-value	**0.001**	**0.009**				** < 0.0001**				**0.001**	**0.006**
Romania	Men (265)	10 (6–16)	100%	-	-	-	79.2%	20.8%	-	-	155 (115–210)	51 (36–73)
	Women (236)	11 (6–17)	100%	-	-	-	74.6%	25.4%	-	-	160 (117–214)	48 (36–68)
	*p*-value	0.3	NA				0.2				0.9	0.2
Spain	Men (402)	4 (2–8)	82.3%	17.7%	-	-	81%	6.5%	7.7%	4.7%	-	-
	Women (244)	6 (2–11)	86.9%	13.1%	-	-	77.9%	5.7%	12.3%	4.1%	-	-
	*p*-value	0.08	0.1				0.2				NA	NA
USA	Men (2704)	4 (1–11)	89.3%	8.9%	-	–	81.4%	8.7%	8.1%	1.7%	107 (76–160)	48 (35–67)
	Women (2440)	4 (1–13)	87.4%	8.6%	-	–	82.2%	8.1%	8%	1.7%	112 (80–165)	48 (38–71)
			***P:0.03***	*P:0.7*								
	*p*-value	**0.03**	** < 0.0001**				0.8				0.3	0.3
Turkey	Men (283)	4 (2–12)	100%	-	-	–	72.8%	14.1%	8.1%	4.9%	140 (100–221)	54 (44–68)
	Women (217)	6 (2–15)	100%	-	-	–	72.8%	17.1%	7.8%	2.3%	135 (110–192)	57 (43–81)
	*p*-value	0.08	NA				0.4				0.7	0.5
Paraguay	Men (258)	6 (3–10)	86.4%	12.1%	1.6%	–	88.5%	11.5%	–	–	180 (195–195)	60 (45–65)
	Women (161)	8.5 (4–12)	89.4%	8.1%	2.5%	–	86.1%	13.9%	–	–	163 (100–190)	65 (41–93)
	*p*-value	**0.01**	0.3				0.5				0.18	0.3

*Median (IQR), *TIA* transient ischemic attacks, *IVT* intravenous thrombolysis, *MT* mechanical thrombectomy

Bold values refer to statistically significant variables

**Table 5:** Stroke outcome in relation to sex among the participating countries

	Sex No	mRS discharge	mRS 3 months
Egypt	Men (2807)	42.4%	54.1%
	Women (1813)	35.2%	47.3%
	*p*-value	**0.001**	** < 0.0001**
Iran	Men (3192)	22.2%	29.6%
	Women (2793)	17.4%	24.3%
	*p*-value	** < 0.0001**	** < 0.0001**
Oman	Men (132)	NA	35.2%
	Women (58)	NA	34.8%
	*p*-value	NA	0.97
Poland	Men (394)	16.1%	17.4%
	Women (260)	47.7%	52.8%
	*p*-value	** < 0.0001**	** < 0.0001**
Romania	Men (265)	46%	56.1%
	Women (236)	39%	48.8%
	*p*-value	0.11	0.3
Spain	Men (402)	-	46.2%
	Women (244)	-	34.1%
	*p*-value	-	0.05
USA	Men (2704)	27.7%	34.2%
	Women (2440)	23.8%	29.9%
	*p*-value	**0.007**	0.1
Turkey	Men (283)	46.3%	60.3%
	Women (217)	31.3%	44%
	*p*-value	**0.001**	** < 0.0001**
Paraguay	Men (258)	-	-
	Women (161)	-	-
	*p*-value	NA	NA

*mRS* Modified Rankin Scale favorable outcome: (< 2)

Bold values refer to statistically significant variables

## Severity

NIHSS was higher in women in all countries except Poland, where men had a severer stroke. Egypt, Iran, the USA, and Paraguay had significantly more severe strokes in women than in other countries.

## Type of stroke

Incidence of ischemic stroke was significantly more in women in Oman and Poland, while it was more in men in the USA. Hemorrhagic stroke was significantly more in men in Oman and Poland.

TIA was more in women in Iran. Moreover, in Egypt and Oman, subarachnoid hemorrhage was similar in both sexes, while other countries did not report it.

As for acute management, in Egypt, only bridging therapy was significantly more often in women; in Oman IVT and MT were more in women, while conservative therapy was more in men so was bridging therapy. Poland reported IVT and bridging more in women, while conservative therapy was more frequently given to men.

Outcome was worse among women in all countries except Poland. This sex difference was significant in Egypt, Iran, USA, and Turkey.

## Discussion

The study of sex differences is an emerging field of stroke epidemiology and care. This study explored sex differences related to various aspects of stroke etiology and management.

### Comparison between sexes in the whole sample

Among the studied cohort of 18,659 patients, stroke incidence was higher among men with a ratio of 1.27:1. And despite that the ratio might vary among studies, yet the incidence is consistently higher among men. Generally speaking, women reportedly have a lower risk of developing stroke than men, though the magnitude of this difference decreases after menopause [8]. In line with this, most of the participating countries reported that age-related difference was reversed for those aged above 60.

Similar to previously published studies, the mean age at stroke presentation was higher among women [9, 10]. On average, women were about 4 years older than men, which is concordant with other research that has also demonstrated that women were older at the time of their first stroke [11, 12].

Regarding the mean age of the whole patients (males and females), we noticed that Egyptian stroke patients were younger than stroke patients in other countries. It is noticeable in most studies from Egypt that the age of stroke patients is younger than other countries. This is possibly either due to multiple risk factors or is genetically determined. The mean age is around 62 in several of our previous research [13, 14].

Regarding risk factor profile according to sex, our findings coincide with other studies [15–18]. Most important to mention is AF that is consistently relevant to sex so female sex has been incorporated as a risk factor in the CHADS2VASc2 score for decision-making about anticoagulation [19].

There was a disparity between our findings and other studies reporting higher prevalence of dyslipidaemia in women [20]. The prevalence of dyslipidaemia was reportedly associated with higher socioeconomic development and is attributable to western diet and high caloric intake [21].

Thus, this low prevalence of dyslipidaemia in our sample could be due to racial differences or dietary habits, given the preponderance of eastern population reaching 61% among our cohort.

On comparison of stroke subtypes, although we found variable patterns between both sexes, yet others reported that with the exception of SAH, there is little evidence of sex difference in stroke subtypes [4, 20, 22].

Regarding stroke severity and outcome, we found that women had a severer stroke on admission and worse outcome which could be attributed to their older age at the time of stroke incidence. However even after adjusting for age, still female sex per se was found to be a determinant for negative outcome [23, 24]. In addition, the poor outcome in women has been ascribed to lower pre-stroke functional status, body mass index, AF, and hypertension [25].

But despite this sex difference in outcome, our results illustrated that the type of management was similar for sexes except for bridging therapy that was more frequently administered in women. Pre-hospital delay was significantly lower among women, while other in-hospital time factors showed no difference. Many previous western studies reported that OTD is longer in women. We suppose that this trend might have changed in the past few years, that is why the title of our article is “Do stroke services still show sex differences? A multicentre study.” We have 2 comments here. First that the bigger number of patients in our study (11,295) is from eastern countries versus (7364) from western countries. Thus, the results might be colored by the eastern culture where elderly women are usually living with caregivers from the family, and any health issue is promptly attended to by family members. This cultural notion was highlighted in a recent reference stating “women are less likely to receive IVT compared to men in the US and most European countries, but not in Asia and Germany.” [26] Second, the trends might be changing due to public awareness of stroke and urgency of early treatment; thus, the gap between sexes is diminishing. This has been mentioned in the same study as the one cited below where they stated “Subgroup analyses including only patients deemed eligible for IVT however did not show any significant sex difference in IVT treatment anymore.” [26].

The sex difference in revascularization therapy varied among various studies. It was previously observed in European and US-based studies but not in studies from Asia and Germany [24, 27].

### Comparison between age groups among women

Concerning stroke subtype, similar to other studies, the presence of cardio-embolic stroke in older patients is explained by higher prevalence of AF [28]. On the other hand, the absence of conventional risk factors explains why younger age group presented with stroke of undetermined/other determined etiology [29, 30].

When it comes to severity and outcome, the presence of comorbidities and premorbid state related to aging, in addition to the higher incidence of cardio-embolic stroke, could explain severer stroke in advanced age [31].

### Comparison of stroke characteristics across participating countries

The median age of women in both Egypt and Oman was younger as compared to all other countries, and this comes in the context of a younger age of stroke for the whole population [23].

The younger age of stroke patients in Egypt and Oman compared to other countries, and on the contrary the very few young patients in Poland, both need further verification by future research.

Also, Poland was the only country to have less stroke severity and better outcome among women, possibly because women had significantly less prevalence of hypertension and less hemorrhagic strokes than men. Moreover, women who had an ischemic stroke were more prone to receive IVT and bridging therapy.

Similar to a previous study [15], there was a trend towards administering interventional management to women that reached a significantly higher level than men in some countries. The shorter onset to door reported for women could be a contributing factor for the type of management.

We can conclude that globally, stroke services provided to women are no more inferior to those provided to men, thanks to previous work that drew attention to this sex disparity. Despite that, still female sex remains an unmodifiable risk factor for severer stroke and worse outcome. Thus, future studies are warranted to explore modifiable female-related risk factors including pregnancy, postpartum, and hormonal changes [8].

## Study limitations

In the current study, all patients were recruited from specialized stroke units where the best acute stroke services are supposedly offered. So, the results do not reflect the state of services in general hospitals in the participating countries.
